# Impact of brand hate on consumer well-being for technology products through the lens of stimulus organism response approach

**DOI:** 10.3389/fpsyg.2022.946362

**Published:** 2022-07-28

**Authors:** Saman Attiq, Abu Bakar Abdul Hamid, Hassan Jalil Shah, Munnawar Naz Khokhar, Amna Shahzad

**Affiliations:** ^1^Air University School of Management, Air University, Islamabad, Pakistan; ^2^Putra Business School, Universiti Putra Malaysia, Seri Kembangan, Malaysia; ^3^School of Social Sciences and Humanities, National University of Science and Technology, Islamabad, Pakistan; ^4^Department of Management Sciences, COMSATS University, Islamabad, Pakistan; ^5^AM MCR Ltd., Manchester, United Kingdom

**Keywords:** self-incongruity, brand hate, brand dissatisfaction, brand retaliation, consumer wellbeing, quality of life, young consumers

## Abstract

Consumer well-being is a micromarketing concept that emphasizes on contributions of marketing activities in social welfare. The major objective of the current study is to analyze the impact of self-incongruence on brand dissatisfaction, brand hate, and consumer well-being. This study has utilized the Self-incongruity Theory and the Stimulus-Organism-Response model to test the impact of self-incongruity on anti-consumption and consumer voice behaviors, and subsequent effects on consumer well-being. Data were collected from young consumers of technology products from major cities of Pakistan. A total of 592 consumers answered a paper-and-pencil questionnaire using purposive sampling technique. The data were analyzed by partial least square structural equation modeling. The findings of this study reveal that functional and symbolic incongruity predict brand hate and dissatisfaction, which is positively related with brand retaliation. Brand retaliation is negatively related with consumer well-being. This study offers implications for product designers, marketers, advertisers and other stakeholders to improve congruence between what young consumers of technology products expect and what brands are offering to mitigate negative attitudes and behaviors and increase consumer well-being.

## Introduction

The prime goal of human activities has always been to attain profound well-being. Researchers from an array of fields such as economics, psychology, and sociology have highlighted the importance of studying human well-being (Diener and Seligman, 2002). Traditionally, marketing research has been focused on achieving consumer delight, developing customer equity (Netemeyer et al., 2004), and encouraging consumers to revisit stores and brands to repurchase and readopt. However, researchers are now shifting their focus from the consumer behavior and brand consumer relationships to explore consumer well-being (Mogilner et al., 2012). A major reason for adoption of the consumer well-being concept is that it not only ignites brand loyalty, but it also influences how consumers make choices in selecting brands and express words of mouth (Schnebelen and Bruhn, 2016). Researchers have also explored determinants of consumer well-being in different stages of consumption (El Hedhli et al., 2016). Some suggested that it is not the material possession but the experience, either ordinary or extraordinary, that brings happiness to older and young people (Bhattacharjee and Mogilner, 2014). Aaker et al. (2011) have elaborated that it is not the money but the time spent with right people and on right activities that will bring happiness. Researchers have also explored the role of consumer personality in bringing happiness.

In brand management literature, only few researchers have focused on how brand experiences, brand–consumer relationship quality, and self-congruence with brands are related with consumer well-being (Schnebelen and Bruhn, 2018). The self-determination theory states that human well-being depends on the achievement of psychological needs of an individual (Ryan and Deci, 2000). In the field of brand-consumer relationship, this implies knowing how brands increase consumer well-being by meeting their customers’ psychological needs. Alternatively, the self-congruity theory states that consumers value brands whose image matches with their own self-image (Sirgy, 1985). There are some studies that have used the self-congruity theory to understand how people idealize what is presented to them by advertising and what is its impact on their well-being (Lou and Tse, 2020). Albrecht et al. (2017) stated that consumers use brands and other possessions to communicate to others their identities and self-image. In this way, they intend to impress others through their specialized possessions or membership to a core group of brand users (Isaksen and Roper, 2016).

Consumers nowadays are cognizant of the fact that brands do have a power to communicate and ignite certain emotions (Isaksen and Roper, 2016) such as brand love (Junaid et al., 2020). For example, users of Nike shirts intend to communicate coolness, whereas users of Apple smartphones indicate their tech-savviness (Chaplin et al., 2014). This fit-in approach of consumers is very important to understand consumer preferences of brands (Albrecht et al., 2017) as well as purchase intention (Akram et al., 2021). This provides a way to understand the importance of “right brand for right consumer.” The right brand does not necessarily mean being the most expensive; rather, the right brand is that which is more socially acceptable and has greater emotional and communicative power to identify with consumers. The identification and match will necessarily be translated into consumer happiness and well-being. Dittmar (2007) argued that material possessions frame individuals’ self-image. Material possessions have a power to influence individual’s beliefs, feelings, and thoughts about their ideal selves and have an impact on compulsive buying. As possessions are vital for formation of consumers’ self-image, any incongruity between expected and real images may lead to negative emotions in consumers (Sirgy, 1985). Salient in negative emotions is brand hate. Brand hate is a less-researched area of consumer behavior (Cherrier et al., 2011). More attention has been paid to positive sides in the area of consumer relationships with brands (Kandampully et al., 2015; Pappu and Quester, 2016). Brand hate is invoked because of many reasons including brand avoidance, anti-consumption (Lee and Ahn, 2016), negative experience (Joshi and Yadav, 2020; Pinto and Brandão, 2021), symbolic incongruity (Banerjee and Goel, 2020), and brand embarrassment (Sarkar et al., 2019).

Researchers have recently shifted their focus to study brand hate in the context of the self-incongruity theory. In this regard, Sirgy and Su (2000) presented an integrative model linking destination environment with self-congruence to see its impact on tourist behaviors. Japutra et al. (2018) also proposed that the self-incongruence felt by consumers is positively related to negative emotions. (Islam et al. (2019) also found that self-incongruency is an important element of Pakistani consumers’ brand hate. Consumers simultaneously check for self-image and product attributes before purchasing a brand. This calls for a wide understanding of consequences of brand hate in relation to self-incongruity. Research studies also suggest that brand hate is sometimes a function of consumer dissatisfaction that arises when brands fail to fulfill idealized characteristics, creating an incongruity between ideal self and self-image (Kucuk, 2018a). Zhang and Laroche (2020) emphasized to study brand hate and other negative emotions, as it may benefit brands by avoiding possible losses due to brand hate and help them increase consumer well-being. Owing to the fact that limited research has been carried out to explore the phenomenon of brand hate (Kucuk, 2018b; Bryson and Atwal, 2019; Pinto and Brandão, 2021), this study intends to evaluate the impact of brand hate on consumer well-being in the light of the self-incongruity theory.

As discussed earlier, brand hate has received little attention from scholars as compared to positive emotions. Evidence from developing countries is further scarce and incomplete. However, research on social psychology considers that marketing efforts that aim to develop self-image of consumers by transmitting materialistic values have a greater influence on poor populations than on rich populations. Chaplin et al. (2014) surveyed 117 children from poor and rich backgrounds. They found that children from poor families were more materialistic than the rich children. This difference was attributed to possibly the lower self-esteem of impoverished children. Poverty is considered to have a negative impact on well-being (Martin and Paul Hill, 2012), as impoverished consumers fail to satisfy their needs. Therefore, such consumers intend to increase their self-identities beyond their status by obtaining material possessions (Hill and Stamey, 1990). Hill et al. (2012) surveyed consumers of 38 countries and compared the behaviors of poor and rich consumers with respect to material possessions. They found that poor consumers belonging to developing countries were more concerned about their material possessions to reconstruct their self-identities. A study noted that the lack of material goods of poor consumers was more powerfully impacting their life satisfaction as compared to their affluent counterparts in other societies. In this way, it may be assumed that consumers from poor developing countries may be more materialistic and sensitive to their possessions and self-image; thus, any incongruity may lead them to display negative emotions such as brand hate. They may express their dissatisfaction and spread negative words of mouth.

Therefore, this study aims to achieve the following objectives:

(1) To examine the impact of self-incongruence (as stimulus) on dissatisfaction and brand hate.

(2) To analyze the influence of dissatisfaction and brand hate (as organism) on retaliation.

(3) To evaluate the impact of retaliation on consumer well-being and subjective well-being (as response).

### Significance

Researchers have acknowledged the positive role of marketing in improvement of consumers’ quality of life (Grzeskowiak and Sirgy, 2008), because consumption is associated with consumer satisfaction that positively enhances their perception of satisfaction with life. However, recent research has focused on more detailed view of quality of life in terms of consumer well-being. More interestingly, many researchers have explored the impact of consumption on positive states, e.g., consumer satisfaction; very little is known about how consumers feel negative emotions when brands fail to meet their stated standards of self-concept.

#### Theoretical significance

This research will be the first of its kind to know how self-incongruity invokes negative emotions and how negative emotions are fatal for brands themselves by dissemination of negative words of mouth by dissatisfied and hating consumers and for consumers themselves in terms of their lowered well-being. This study specifically contends that consumers’ perception of self-incongruity, which results from a perceived mismatch between self-concept and product concept, acts as a stimulus to produce psychological reactions (as an organism), such as feelings of dissatisfaction and brand hate, which eventually lead people to engage in retaliatory behavior and harm their well-being (as response) (Attiq et al., 2017). Furthermore, this study also integrates the self-congruity theory with the stimulus organism response (SOR) model. In this way, it is argued that consumers’ perceptions of self-incongruence will function as a stimulus to develop negative attitudes and behaviors such as dissatisfaction and brand hate, which will further result in consumer retaliation (Jabeen et al., 2022). This whole sequence will ultimately affect consumers’ well-being as well as their subjective well-being.

#### Contextual significance

Researchers have identified the relevance of the self-congruity theory in the food sector. Islam et al. (2019) that found functional and symbolic congruity was a major reason of brand hate in young consumers of fast-food products in Pakistan. They also found a role of self-image and brand attributes in translating the effects of congruence on brand hate. Previously, a study conducted on Chinese restaurants found that consumer perceptions of physical environment and image were positively related to consumer intention and attitudes (Ryu et al., 2012). However, another study also found a positive association between restaurants’ overall image and consumer attitudes (Han and Hyun, 2017; Akram et al., 2020). Although previous studies have tested the role of congruence in consumer satisfaction, this study contrarily intends to unearth the role of incongruence in emergence of negative attitudes such as consumer dissatisfaction and brand hate in technology-related products rather than in the food sector.

#### Practical significance

Since consumer satisfaction also has an impact on consumer well-being, product designers need to focus more on functional aspects of technology brands to satisfy consumers. Young consumers of technology-related brands are not only more aware of people but also more confident and independent decision-makers. Corporate managers must therefore realize that the right brand for the right consumer is of paramount importance, particularly in technology-related brands. Marketers who emphasize on consumer well-being (quality of life marketers) need to focus on creating brand communities to facilitate self-concept and enhance consumer well-being (Grzeskowiak and Sirgy, 2008). Marketers of technology brands also need to focus on technology brand communities by engaging young consumers to become ambassadors of their brands. Brands will have to sponsor major youth-related activities in universities and colleges by holding engaging activities, sponsoring their sports and academic events, and sharing some souvenirs and gifts ascribed with logos and messages to create a connection between them and consumers.

### Theoretical background

#### Integration of self-incongruity theory and stimulus organism response model

The self-congruity theory is rooted in the understanding of self-concept and is helpful in understanding and predicting consumer behavior. It refers to a psychological process and a consequence where consumers engage in comparison between brand image and their own self-concept (Sirgy, 2018). This theory measures the match between consumer’s self-concept and his image about a brand. Consumer self-concept refers to the person’s overall image about self, considering themselves as an object (Rosenberg, 1979). Sirgy (1985) proposed 4 components for a framework of self-concept, namely, actual self-, ideal self-, social self-, and ideal self-image. Actual self-image refers to consumers’ true perceptions of themselves and personal identity. Ideal self-image is what consumers aspire to become (Sirgy, 2018; Gómez-Rico et al., 2021). It also reflects the attributes a consumer wishes to acquire. Social self-image, on the contrary, refers to a consumer’s belief about how significant others see themselves, in other words, how people view him/her based on certain characteristics. Lastly, ideal social image reflects how consumers want to be seen and dealt with by others (Sirgy, 2018; Sop, 2020). These four components of the consumer self-concept are induced while making preferences about brands in the market. The second aspect of the self-congruity theory is brand-user image or brand personality. Brand personality refers to the consumers’ perceptions of brands having distinct traits such as excitement, sincerity, competence, ruggedness, and sophistication (Sirgy, 2018; Sop, 2020). Consumers match their self-image with brand personality while making a purchase decision. For example, some brands such as Apple may be considered by consumers as creative, artistic, and so forth (Roggeveen et al., 2021). The self-congruity theory affects consumer pre-purchase behaviors, influencing their choices of brands and post-purchase behaviors such as satisfaction or dissatisfaction with brands. Self-congruity has a significant relationship with dissonance and negative emotions (Sirgy, 2018), brand personality and image (Wu et al., 2020), consumers’ post-purchase behavior, and higher level of consumer satisfaction, trust, and brand love (Klabi, 2020; Tran et al., 2021).

Conversely, self-incongruity will result in opposite behaviors such as brand hate, negative words of mouth, dissatisfaction with a brand, and brand revenge. The self-congruity framework is taken from the seminal study of Sirgy and Su (2000) who presented a self-congruity model in the hospitality sector. They found that visitors’ travel behaviors are dependent on the destination image and perceptions of self-congruity. There are important reasons to use the self-incongruity theory in this study. This theory has been adopted in various consumption-related studies including on the food sector (Shamah et al., 2018). Usakli and Baloglu (2011) considered self-congruity as a phenomenon that extends person’s views about self. Researchers contend that self-concept is amplified by consumption such that people use brands and products that are congruent with self-image. In this way, marketers use the self-congruity theory as an important mechanism to understand and influence the consumption patterns of consumers; especially, the success and failure of brands are mostly attributed to the self-congruity theory (von Mettenheim and Wiedmann, 2021).

This study also uses the SOR model to explain its research framework. This model pertains to cognitive psychology and provides greater basis for understanding of consumer behavior. The basic tenet of this theory states that consumer behavior is the function of some external stimulus and internal processing (Mehrabian and Russell, 1974; Jacoby, 2002). The influence of external stimulus combined with internal and psychological elements impacts behavior. This model has been popularly used in environmental psychology in which certain aspects of environment function as stimulus (S) to have an influence over person’s internal feelings (O) and eventually result in a behavior (R) (Eroglu et al., 2001). The stimulus in the SOR model refers to factors in the environment surrounding consumers and arousing consumer emotions (Jacoby, 2002). In connection to the self-congruity theory, any incongruence felt by consumers with regard to product concept and self-concept will ignite certain action-oriented emotions such as brand hate and dissatisfaction (Kamboj et al., 2018; Khatoon and Rehman, 2021).

### Consumer well-being

Traditionally, marketers and scholars were more interested in understanding consumer satisfaction, which was thought to be a precursor to consumer loyalty, repurchase intention and so on. Consumer satisfaction reflects the emotional or cognitive response of a consumer related to particular product based on expectation and experience during a particular period or point of time (Giese and Cote, 2000). Although consumer satisfaction has received greater attention from scholars and practitioners alike, this concept is limited to experience and consumption of a product itself and resultant consumer behaviors such as loyalty, repurchase intention, market share, and increasing sales (Sirgy et al., 2007). However, an alternative concept of consumer well-being goes beyond merely consumer satisfaction as it adds quality of life with consumer satisfaction. More specifically, the concept of consumer well-being is based on the assumption that increase in consumer well-being will not only increase consumer satisfaction but will yield greater levels of life satisfaction, decrease ill-being, and increase social welfare and life happiness (Sirgy et al., 2007). Consumer well-being is part of a general stream of studies that unearthed the impact of marketing on consumers’ quality of life (Sirgy et al., 2007). On the other hand, a recent study (Kuanr et al., 2021) found an impact of well-being on brand avoidance.

Thus, CWB intends to measure consumers’ well-being implicitly and explicitly by establishing links between consumer satisfaction and happiness. Generally, researchers also consider CWB as a state of mind that reflects consumer satisfaction and pleasure obtained from consumption of products (Veenhoven, 2003; Lee and Ahn, 2016). Fang et al. (2014) defined CWB as a process that aligns individual needs with societal needs such as psychological, physical, social, and economic needs. Zhao and Wei (2019) explained consumer well-being as an overall cognitive and emotional consumer response with regard to the consumption process considering three major aspects: satisfaction of consumers with a product, their positive emotional response, and quality of life. Authors argue that consumer satisfaction is key to their well-being. As they consume products, they fulfill their various needs such as material needs and social and psychological needs. This needs satisfaction, as per the self-determination theory, it provides them happiness. Second, the happiness derived from consumer satisfaction brings positive emotions among consumers. Yang and Galak (2014) suggested that consumer well-being is not limited to functional aspects and value but that it is also viewed from the emotional value that consumption of products brings to consumers. For example, when consumers consume food, it not only provides a functional value but the delicacy of food makes consumers happy and delighted, resulting in positive emotions (Apaolaza et al., 2018). These positive emotions of consumption of delicious foods are essential part of consumer well-being. The third aspect attached to consumer well-being is the feeling of quality of life. Grzeskowiak and Sirgy (2008) indicated that consumer well-being is reflected by their perception of improvement in quality of life as a consequence of consumption of products and services. Research also supports this notion as consumers of superstores feel higher levels of quality of life while shopping through stores (Grzeskowiak et al., 2016). Furthermore, Hollebeek and Belk (2021) studied well-being and consumer engagement in the context of technology.

### Dissatisfaction

Dissatisfaction will arise as long as consumer expectations are higher than actual experience following the use of a product or a service. Dissatisfaction with a brand’s performance can be attributed to a mismatch between expectations and results (Oliver, 1981). Disconfirmation, which can be positive or negative, is defined by this gap as either positive or negative depending on whether the performance meets or exceeds expectations (Van Ryzin, 2013). Consumer dissatisfaction behavior has been identified by a number of researchers. It is self-evident that a consumer’s decision to repurchase a product or service is heavily influenced by previous encounters with the same product or service (Ferguson and Johnston, 2011). A growing proportion of customers would be dissatisfied with a company when they have had multiple negative experiences with it, and, more importantly, when they believe that the quality of a product or a service is constantly deteriorating (Boadi et al., 2017). When customers are displeased with a product or a service, they get extremely emotional and display a variety of behaviors such as regret and dissatisfaction (Jang and Kim, 2011). In accordance with Oliver’s (1981) expectancy-disconfirmation theory, consumers are said to be dissatisfied with a brand’s performance when their expectations are not met. As a result, customers are dissatisfied with a brand’s messages and offerings, which leads to brand dissatisfaction (Anaza et al., 2021).

### Brand hate

Defining brand hate in a broader sense is necessary, as the term encompasses a wide spectrum of unpleasant feelings (Kucuk, 2019a). Thus, the concept is defined as: “consumers’ detachment from a brand and its associations as a result of consumers’ intense and deeply held negative emotions” (Kucuk, 2019b). Negative consumer-brand connections have been widely discussed in the literature that either focuses on products or does not distinguish between services and products (Knittel et al., 2016; Davvetas and Diamantopoulos, 2017; Curina et al., 2020). There have been limited studies that have explored brand hate in the context of customer service (Curina et al., 2020). Brand hate is a relatively new and understudied phenomenon in the field of negative customer feelings (Bryson and Atwal, 2019). Because of the less attention paid, Brand hate was once assumed to be the reverse of brand love (Palusuk et al., 2019; Gumparthi and Patra, 2020). According to the literature on psychology, contempt, hate, and other negative emotions cover anger and other negative feelings (Sternberg, 2003). Research on branding has called for deeper investigation on the negative emotions felt by consumers as they go through the purchasing process (Kucuk, 2019b). First, to explore the significance of negative customer-brand interactions, Park et al. (2010) and Fournier and Alvarez (2013) urged greater research on this area. Second, earlier research has shown that customers form anti-brand communities to discuss and share their bad feelings and experiences with certain brands, as well as tactics for dealing with the disliked brands (Hollenbeck and Zinkhan, 2010; Tuhin, 2019). Brands that are well-liked and well-recognized are particularly vulnerable to this type of consumer behavior (Bryson and Atwal, 2019). Third, while going through a bad experience with a service provider might lead to negative feelings such as brand hate, marketing literature shows how this can happen (Hegner et al., 2017). Businesses and their associated brands suffer as a result of such negative emotions (Mannan et al., 2017).

### Incongruity, dissatisfaction and brand hate (stimulus-to-organism)

Soscia (2007), using cognitive theory of emotions, contended that consumers’ feeling of goal incongruence was related to their feeling of sadness and anger. These negative emotions were then positively related to complaining behavior and negative words of mouth. Zarantonello et al. (2018) found image incongruence as a major trajectory of consumer brand hate. The concept of symbolic congruence entails that consumers judge their brands in terms of personality traits and match them with their own personality. In this way, brands that are congruent with their personality traits are valued and adopted more often (Wu et al., 2020). Brands that are congruent with consumer personalities will develop social patterns that enable them to feel intimacy with the brands (Kressmann et al., 2006). However, any incongruity will lead consumers to display negative emotions (Sung et al., 2020). Research suggests that consumers’ buying behavior is not only dependent on satisfaction of basic needs but what a product is representing or a meaning it is conveying is also an important factor in consumer buying decision (Hosany and Martin, 2012). Elliott (1997) described symbolic congruence as a notion that consumers no longer consider the material benefits offered by brands as a basis for their decision; rather, they consider the symbolic meaning protrayed by brands in terms of associated brand image, reputation, and personality attributes as an important factor (Bakari and Bakari, 2019). Futhermore, Sirgy and Su (2000) explained the self-congruity theory as the fit between what consumers consider of themselves (self concept) and what an image brand enjoys (brand image). Rosenberg (2017) defines self-concept as a holistic view of consumer about himself or herself considering himself or herself as an object. Recently, a research study on brand management has found the consumer feeling of symbolic incongruity to be positively related to negative emotions like brand hate (Hegner et al., 2017). A recent research study conducted on anti-apple consumers found that symbolic incongruity, along with triggers such as brand inauthenticity, incompatibility of ideology, and previous unfavorable experience, is positively related to brand hate (Rodrigues et al., 2020).

Hypothesis 1: Symbolic incongruity has a significant and positive impact on dissatisfaction.

Hypothesis 2: Symbolic incongruity has a significant and positive impact on brand hate.

Unlike symbolic congruence, which is a match between the image of a brand and consumer self-concept, functional congruence reflects how a product’s utilitarian attributes match with consumers’ expected attributes (Sirgy et al., 1991; Wu et al., 2020). In other words, functional congruence reflects product attributes that consumers idealize and wish to seek into a product. It is because consumers’ perspective product quality prefers the choices made by the consumers (Griffith and Lee, 2016). Researchers found that functional congruence predicts different consumer behaviors such as preference of brands and attitudes toward brands. Sirgy and Su (2000) expressed functional congruence as a set of product attributes and found it positively related to individual expectations on product performance. They further explored utilitarian congruence in the context of the hospitality industry. They found that peoples’ perception of utilitarian congruence was related to perception of food quality, food price, and service quality. Sirgy et al. (1991) concluded that compared to self-congruence, functional congruence is proved to better predict consumer behavior (Wu et al., 2020). In the literature on product failures, there are many determinants explored by previous research such as increased prices, unfavorable store environment, and poor product quality, which foster negative emotions among consumers and may lead to brand hate (Krishnamurthy and Kucuk, 2009; Zarantonello et al., 2016; Hegner et al., 2017). The literature suggests that functional incongruence increases customer dissatisfaction based on comparison of product quality and store environment. Hegner et al. (2017) found a positive link between functional incongruity and brand hate. Research also suggests that unmet expectations and disconfirmed beliefs create dissatisfaction, which is related to negative words of mouth. Therefore, we hypothesize the following:

Hypothesis 3: Functional incongruity has a significant and positive impact on dissatisfaction.

Hypothesis 4: Functional incongruity has a significant and positive impact on brand hate.

### Brand retaliation

Brand retaliation is defined as consumers’ actions directed toward punishing a brand in response to a consumer feeling that the brand has betrayed them or caused any damage to them (Bechwati and Morrin, 2003). This reflects a provoked approach of consumers as a coping strategy when consumers have a strong intention to get revenge from the brand by engaging in revengeful and rude behaviors because of the felt transgression (Jabeen et al., 2022). Consumer retaliation is necessarily a consumer response directed at bringing a brand down to some extent to balance the perceived damages (Grégoire and Fisher, 2008).

Consumer retaliation takes different shapes and forms. Retaliation may take the form of bitter complaining in the shape of a consumer abusive behavior toward frontline employees (Hibbard et al., 2001); other forms include more aggressive ways such as vandalism and physical aggression (Huefner et al., 2002). Consumers may retaliate indirectly such that instead of engaging with brands and employees directly they tend to share their bad experiences to their family, friends, and other acquaintances aimed at sharing negative information and causing damage to the brand (Lee et al., 2020).

### Dissatisfaction, brand hate and brand retaliation (organism–to-response)

Consumers who feel dissatisfaction also consider themselves as having been betrayed. This feeling of betrayal leads to anger, a strong negative emotion that leads consumers to retaliate strongly and negatively in the form of spreading negative information (Tripp and Grégoire, 2011). These feelings of anger and betrayal foster intention to have revenge from the brands (Lee et al., 2013). Lee et al. (2020) concluded in their research that if consumers feel that they are betrayed because of unfairness of a brand, they feel animosity toward the brand. The animosity leads them to retaliate against that brand. Chang et al. (2015) also argued that when consumers are dissatisfied with a brand, they engage in complaining behavior (p. 50). Researchers have been interested to know what causes consumer retaliation. Major precursors to consumer retaliation have been consumer dissatisfaction with a brand prompted by service failures or any mismatch of expectations and performance (Grégoire and Fisher, 2008). Previously, research has been focused on passive forms of consumer expressions of their negative experiences such as exiting the relationship with brands or just filling the complaint box. However, consumers’ active and aggressive behaviors such as engaging in physical assaults, insulting frontline officers, using offline and online platforms to share negative words of mouth, and vandalizing properties are also gaining the attention of scholars (Grégoire et al., 2010). Moreover, recent studies have found that when consumers feel incompatibility between what they think of the products and what others view, it will lead consumers to boycott the products (Ferguson and Johnston, 2011). A very recent study that collected data from users of online food platforms in the United States using the SOR model found that consumer dissatisfaction and brand hate positively impact consumer retaliation (Jabeen et al., 2022). A study from Pakistan also found a positive association between brand hate and brand retaliation (Noor et al., 2021). Based on the above analysis, this study hypothesizes that:

Hypothesis 5: Dissatisfaction has a significant and positive impact on brand retaliation.

Hypothesis 6: Brand hate has a significant and positive impact on brand retaliation.

### Brand retaliation and consumer well-being

Consumer well-being is viewed as fulfillment of individual needs and expectations related to consumption patterns (Burroughs, 2012). Researcher such as Anderson et al. (2013) suggested that when consumers feel betrayed and dissatisfied, their feeling of happiness decreases, retarding their well-being. Research also suggests that discontented consumers are likely to engage in sabotaging behaviors such as boycotting of brands and sharing negative information to others using different media platforms, thus decreasing their well-being and happiness (Makarem and Jae, 2016; Ali, 2021).

Morawczynski and Pickens (2009) argued that complaining consumers make fewer visits to stores to show their anger and dissatisfaction with a brand. Consumer satisfaction refers to totality of consumers’ affective and cognitive reactions to services, products, and experiences related to brands (Giese and Cote, 2000). Any bad experience that surpasses the expected norms will lead to consumer dissatisfaction. That is why marketers and retailers have long been interested in taking care of consumer satisfaction/dissatisfaction as it is strongly linked with consumers happiness (Kim and Boo, 2011). Consumer satisfaction has long been the ultimate goal of retailers because of its powerful influence on purchase intention, repurchase, brand loyalty, word-of-mouth, and sales. Otieno et al. (2005) found in their study on women consumers of clothing from the United Kingdom that consumers who were dissatisfied with a variety of features related to purchase of brands reported their general unhappiness and discontent. This implies that dissatisfaction with brands decreases consumer well-being (Grzeskowiak et al., 2016).

**FIGURE 1:**
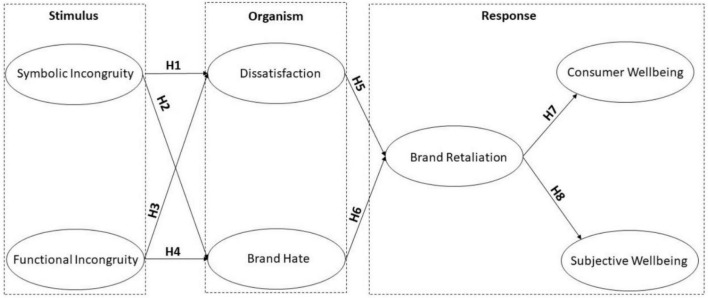
Conceptual framework.

Apart from consumer well-being, marketing researchers have been interested to know the relationship between subjective well-being and consumer experiences related to marketing activities and consumption (Ganglmair-Wooliscroft and Lawson, 2011). This essentially means that consumer experiences of brands have an association with subjective well-being. As subjective well-being is such a broad concept, it lacks precise meaning and definition. However, it has been used in a variety of contexts, and its relative meaning is also described within the purview of that context (Ares et al., 2014). Although empirical studies testing direct relationships between brand retaliation and subjective well-being are scarce, a recent research study unearthed a role of subjective well-being on brand avoidance. More specifically, the study found that brands that violate consumer-perceived ethical norms experienced subversion by brand avoidance by consumers who have high regard for subjective well-being (Kuanr et al., 2021). Based on the above argumentation, this study hypothesizes that brand retaliation will, as a result of negative consumer experiences and negative emotions such as brand dissatisfaction and brand hate, decrease consumer subjective assessment of well-being (see Figure 1).

Hypothesis 7: Brand retaliation has a significant and negative impact on consumer well-being.

Hypothesis 8: Brand retaliation has a significant and negative impact on subjective well-being.

## Materials and methods

### Data collection

Since a survey is conducted to measure behaviors and evaluate relationships between variables and constructs (Newsted et al., 1998), primary data from the respondents were obtained by survey using a quantitative method. Furthermore, in social sciences, survey design has been used extensively in measuring behaviors. Data were collected from users of technology devices (i.e., smartphones, laptops, and audio visual products) by paper-and-pencil survey in shopping centers, malls, and universities in Pakistan’s major cities (i.e., Islamabad, Rawalpindi, Karachi, Multan, and Lahore) where individuals from all over the country reside to seek jobs and education. A focus group was performed with three smartphone and tablets users and three research scholars (with specialization in marketing) to establish content validity and improve the instrument. Before finalizing, the questionnaire was pilot-tested to confirm its validity and reliability. A total of 50 respondents (technology device users) were included in the pilot study; however, they were later omitted from the final data analysis. The results of the pilot study showed that majority of the measurement scales were valid. Undesirable scales were subsequently altered. Purposive sampling was conducted to target individuals with specific characteristics that were relevant to the study’s objectives (Etikan et al., 2016). The researchers guaranteed the respondents’ privacy. To further minimize social desirability bias, we emphasized that there are no wrong or write responses (Randall and Gibson, 1990). The survey was conducted in English for a host of reasons. Based on the researchers’ anecdotal experience, a considerable number of target audiences of technology device users in Pakistan are comprised of urban residents who are well-educated and employed in multinational companies where English proficiency is required. Resultantly, English communication is not a big concern for these users. Previous findings have also corroborated the fact that English is commonly spoken in Pakistan (Kashif and Khattak, 2017; Islam et al., 2019). Out of the 800 surveys sent, 593 people responded. After eliminating missing values, the final dataset contained 567 responses, resulting in a 70.8% response rate. Table 1 shows the demographic details of the participants.

**TABLE 1:** Sample characteristics.

Demographic	Category	Frequency (Percentage)
Gender	Male	363 (64)
	Female	204 (36)
Age (In years)	Less than 20	112 (19.8)
	21–30 years	406 (71.6)
	31–40 years	23 (4.1)
	40 years and above	26 (4.6)
Education	Intermediate	88 (15.5)
	Bachelors	349 (61.6)
	Masters	108 (19)
	Doctorate	3.9)

### Measurement scales

All of the constructs have been tailored and conceptualized using established scales. The construct items were based on measurement scales from a different source, and the responses were recorded on a five-point Likert scale ranging from 1 (strongly disagree) to 5 (strongly agree). Symbolic incongruity is measured through five items adapted from Sirgy et al. (1991). The sample item of symbolic incongruity is “the products of brand X do not reflect who I am.” Functional incongruity is measured through six items adapted from Sirgy et al. (1991). The sample item of functional incongruity is “the product price is high.” Brand hate is measured through six items taken from Lee et al. (2009). The sample item of brand hate is “I hate brand X.” Brand retaliation is measured through five items adapted from Hegner et al. (2017). The sample item of brand retaliation is “I have deliberately bent or broken the policies of the brand.” Consumer well-being is measured through four items adapted from El Hedhli et al. (2013), and the sample item is “this brand satisfies my shopping needs.” Subjective well-being/happiness is measured through three items adapted from Lyubomirsky and Lepper (1999), and the sample item is “compared to most of my peers, I consider myself happy.” Subjective well-being/overall life satisfaction is measured through five items adapted from Pavot and Diener (2008), and the sample item is “I am satisfied with life.”

### Data processing procedure

This study employed the Partial Least Square–Structural Equation Modeling (PLS-SEM) technique to investigate the proposed model. Because of its capacity to combine linear regression with confirmatory factor analysis, PLS is quite popular and widely used. In comparison with covariance-based structural equation modeling, PLS is also accurate in finding actual paths and does not detect non-existent paths (Goodhue et al., 2012). This technique is intended to be used in development and investigation of complex relationships between various variables. Furthermore, this technique is beneficial in studies on hypotheses as well as association between various variables. It also aids in investigation of causal relationships between latent variables. The sample size for testing structural models with PLS should be at least 10 times the number of independent variables in the model. This condition was met, because the current model comprised five independent variables (consumer well-being and subjective well-being). The PLS-SEM model is divided into two steps. The first model is referred to as “measurement model” and the second model is referred to as “structural model.” The two models use various ways to validate the research model (Vinzi et al., 2010).

## Results

### Respondent profile analysis

A total of 567 people took part in the study, with 363 (64%) of them being male technology device users and 204 (36%) being female technology device users. Since the information was gathered from technology device users, the level of education showed that 15.5 percent of the respondents have an intermediate level education. Table 1 shows that 61.6 percent of the people have a graduate-level degree, 19 percent has a master’s degrees, and that only 3.9 percent has a doctoral degree.

### Measurement model

For structural equations, the current study used the PLS (partial-least-squares) modeling technique. The measurement model’s significance is increased by confirming the observed constructs and their associated items. In the measurement model, the outer loads are first assessed. Based on the criterion, items with outer loadings of less than 0.7 are excluded (Hair et al., 2019). The data fit this condition, as shown in Table 2.

**TABLE 2:** Results of the measurement model.

Constructs	Code	Outer loadings	Cronbach’s alpha	Composite reliability	Average variance extracted
Symbolic incongruity	SYI1	0.86	0.89	0.92	0.71
	SYI2	0.83			
	SYI3	0.84			
	SYI4	0.82			
	SYI5	0.84			
Functional incongruity	FUI1	0.81	0.88	0.91	0.63
	FUI2	0.82			
	FUI3	0.74			
	FUI4	0.77			
	FUI5	0.81			
	FUI6	0.80			
Dissatisfaction	DIS1	0.87	0.91	0.93	0.78
	DIS2	0.89			
	DIS3	0.89			
	DIS4	0.88			
Brand hate	BRH1	0.83	0.91	0.93	0.70
	BRH2	0.85			
	BRH3	0.85			
	BRH4	0.85			
	BRH5	0.82			
	BRH6	0.80			
Brand retaliation	BRR1	0.83	0.89	0.92	0.70
	BRR2	0.83			
	BRR3	0.87			
	BRR4	0.83			
	BRR5	0.80			
Consumer wellbeing	CWB1	0.85	0.90	0.93	0.77
	CWB2	0.90			
	CWB3	0.88			
	CWB4	0.87			
Overall life satisfaction (subjective wellbeing- component 1)	LST1	0.83	0.92	0.94	0.75
	LST2	0.88			
	LST3	0.90			
	LST4	0.86			
	LST5	0.86			
Happiness (subjective wellbeing- component 2)	HAP1	0.89	0.89	0.94	0.82
	HAP2	0.90			
	HAP3	0.91			

After a thorough examination of the outer loadings, the next step is to verify the reliability and validity of all the constructs. Two key metrics for evaluating reliability are composite reliability and Cronbach alpha. All of the Cronbach alphas must be at least 0.7 (Hair et al., 2017) (refer to Table 2). In the same way, all the composite reliability values must be at least 0.7 (Hair et al., 2017) (refer to Table 2). The average variance extracted (AVE) is evaluated for convergent validity testing of the variables. All of the AVE values must be at least 0.5 (Fornell and Larcker, 1981) (refer to Table 2). As a result, we concluded that the data had sufficient convergent validity among the latent constructs.

Finally, the discriminant validity of all the research variables is evaluated. This is performed using the heterotrait-monotrait (HTMT) method of testing discriminant validity. The HTMT score should be less than 0.85 (Henseler et al., 2015). Table 3 reports the findings and demonstrate the presence of the discriminant validity (refer to Table 3).

**TABLE 3:** Discriminant validity/HTMT ratio.

	**SYI**	**FUI**	**DIS**	**BRH**	**BRR**	**CWB**	**LST**	**HAP**
Symbolic incongruity								
Functional incongruity	0.74							
Dissatisfaction	0.76	0.71						
Brand hate	0.80	0.81	0.76					
Brand retaliation	0.66	0.74	0.66	0.68				
Consumer wellbeing	0.75	0.77	0.75	0.77	0.77			
Overall life satisfaction	0.73	0.72	0.70	0.69	0.77	0.79		
Happiness	0.74	0.73	0.74	0.72	0.75	0.78	0.78	

SYI, symbolic incongruity; FUI, functional incongruity; DIS, dissatisfaction; BRH, brand hate; BRR, brand retaliation; CWB, consumer well-being; LST, overall life satisfaction; HAP, happiness.

### Structural model

**FIGURE 2:**
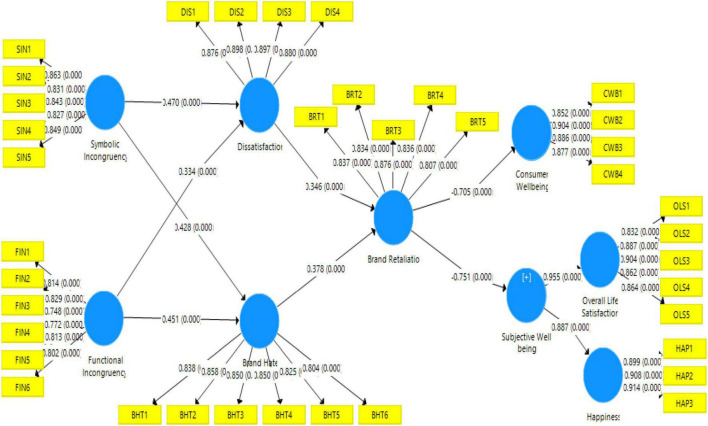
Structural model.

The structural model was tested for research hypotheses among the constructs after the measurement model was examined (see Figure 2). Hair et al. (2017) proposed five criteria for evaluating structural models: multi-collinearity, hypothesis importance, *R*^2^ evaluation, *f*^2^ evaluation, and *Q*^2^ evaluation. For all the constructs, a variance inflation factor test was conducted as the initial step in determining multi-collinearity. The VIF value was less than 3.3, which is Hair et al.’s (2017) recommended threshold and indicated that there was no concern about multi-collinearity (refer to Table 4).

**TABLE 4:** Collinearity test.

	SYI	FUI	DIS	BRH	BRR	CWB	LST	HAP	SWB
Symbolic incongruity			1.84	1.84					
Functional incongruity			1.84	1.84					
Dissatisfaction					1.95				
Brand hate					1.95				
Brand retaliation						1.00			1.00
Consumer wellbeing									
Overall life satisfaction									
Happiness									
Subjective wellbeing							1.00	1.00	

SYI, symbolic incongruity; FUI, functional incongruity; DIS, dissatisfaction; BRH, brand hate; BRR, brand retaliation; CWB, consumer well-being; LST, overall life satisfaction; HAP, happiness; SWB, subjective well-being.

Then, the proposed hypotheses were tested (refer to Figure 2). Symbolic incongruity (β = 0.47, *p* < 0) had a significant effect on dissatisfaction, which supported H1. H2 was also supported as symbolic incongruity had a significant effect on brand hate (β = 0.42, *p* < 0). Functional incongruity had a significant effect on dissatisfaction (β = 0.33, *p* < 0), which supported H3. In the same way, H4 was also supported as functional incongruity (β = 0.45, *p* < 0) had a significant effect on brand hate. Dissatisfaction had a significant effect on brand retaliation (β = 0.34, *p* < 0); thus, H5 was supported. H6 was also supported as brand hate had a positive impact on brand retaliation (β = 0.37, *p* < 0). Brand retaliation had a negative effect on consumer well-being (β = -0.70, *p* < 0); therefore, H7 was supported. Finally, brand retaliation also had a negative effect on subjective well-being (β = -0.75, *p* < 0), which supported H8. The results are shown in Table 5. The model’s prediction accuracy was then measured using *R*^2^ (refer to Table 6).

**TABLE 5:** Analysis of the hypotheses.

Structural paths	β	*p*-value	Results
H1: Symbolic incongruity → Dissatisfaction	0.47	0.00	Supported
H2: Symbolic incongruity → Brand hate	0.42	0.00	Supported
H3: Functional incongruity → Dissatisfaction	0.33	0.00	Supported
H4: Functional incongruity → Brand hate	0.45	0.00	Supported
H5: Dissatisfaction → Brand retaliation	0.34	0.00	Supported
H6: Brand hate → Brand retaliation	0.37	0.00	Supported
H7: Brand retaliation → Consumer wellbeing	–0.70	0.00	Supported
H8: Brand retaliation → Subjective wellbeing	–0.75	0.00	Supported

**TABLE 6:** Coefficients of determination (*R*^2^).

Constructs	*R* ^2^
Dissatisfaction	0.54
Brand hate	0.64
Brand retaliation	0.44
Consumer wellbeing	0.49
Subjective wellbeing	0.56

Effect size (*f*2) is calculated in the fourth step. Hair et al. (2017) recommended that *f*2 values above 0.02 represent a weak effect size, values above 0.15 represent a moderate effect size, and values above 0.35 represent a strong effect size. The results are displayed in Table 7.

**TABLE 7:** Effect sizes (*f*^2^).

	SYI	FUI	DIS	BRH	BRR	CWB	LST	HAP	SWB
Symbolic incongruity			1.84	0.31					
Functional incongruity			1.84	0.28					
Dissatisfaction					0.11				
Brand hate					0.13				
Brand retaliation						0.99			1.29
Consumer wellbeing									
Subjective wellbeing							10.28	3.67	

SYI, symbolic incongruity; FUI, functional incongruity; DIS, dissatisfaction; BRH, brand hate; BRR, brand retaliation; CWB, consumer well-being; LST, overall life satisfaction; HAP, happiness; SWB, subjective well-being.

Finally, a blindfolding procedure was used to assess predictive relevance (i.e., *Q*^2^). The results met the recommended criterion of Hair et al. (2018), i.e., *Q*^2^ > 0. The results are displayed in Table 8.

**TABLE 8:** Blindfolding analysis (*Q*^2^).

Constructs	*Q* ^2^
Dissatisfaction	0.42
Brand hate	0.45
Brand retaliation	0.30
Consumer wellbeing	0.38
Subjective wellbeing	0.37

## Discussion

This study aimed to test the determinants of consumer well-being. More specifically, it tested the impact of self-congruity measured in terms of functional and symbolic incongruities on the development of negative attitudes and emotions such as brand dissatisfaction, brand hate, and, consequently, brand retaliation. This study utilized the self-congruity theory and the SOR model to guide its framework and underlying mechanism among variables. A detailed discussion of the hypotheses is given below.

This study found a positive impact of symbolic incongruity on brand hate and brand dissatisfaction. The findings imply that brands that are not thought to be having personality traits similar to those of consumers’ own personality are not liked by consumers. In other words, consumers’ evaluation of brand personality resulting in any mismatch will end up in development of brand hate. This finding supports the notion of impact of “identity clashes” on negative consumer emotions (Kucuk, 2019a). Kucuk (2019a) argued that when consumers find a mismatch between brand personality and consumers’ own personality, they tend to hate a brand. Malär et al. (2011), in two empirical studies involving 167 brands and more than 2,000 consumers, found that consumers’ lack of actual self-congruence with brand personalities was positively related to emotional attachment with brands. A recent research study conducted on anti-apple consumers found that symbolic incongruity along with triggers such as brand inauthenticity, incompatibility of ideologies, and previous unfavorable experiences are positively related to brand hate (Rodrigues et al., 2020). A study on the Pakistani food sector also found a positive association between symbolic incongruence and brand hate (Islam et al., 2019). A very recent study on the telecom sector of Portugal found that consumers who have high regard for symbolic incongruity showed greater levels of brand hate (Pinto and Brandão, 2021).

The third and fourth hypotheses were related to the impact of functional incongruity on brand dissatisfaction and brand hate. It was hypothesized that functional incongruity, referred to as consumers’ perception of whether products’ attributes match consumers’ expected attributes, will be positively related to brand dissatisfaction and brand hate. The results of this study support the hypotheses. The findings reflect functional incongruence as a major stimulus for consumers’ negative attitudes such as dissatisfaction and brand hate (Chon and Olsen, 1991; Islam et al., 2019). Researchers have always talked about consumers’ preferences and likely attitudes toward utilitarian aspects of products, and this study supports the notion that the functional aspects are still important for shaping consumers behaviors and emotions (Kohli et al., 2021). This is even true in technological products. As tech products are especially designed and valued for their functional sophistication, any failure of such brands on functional aspects will stimulate negative emotions and attitudes such as brand hate and brand dissatisfaction (Wu et al., 2020). Functional congruity is also important in services that keep technology as a core medium to connect with customers such as internet banking and telecommunication. Any incongruity between expected modes of services and actual services will ignite negative consumer emotions and make customers dissatisfied (Tavera-Mesias et al., 2021).

Hypotheses 5 and 6 were related to the impact of brand dissatisfaction and brand hate on brand retaliation. The results supported the hypotheses. The findings imply that dissatisfied consumers take revenge on hated brands by engaging in anti-brand activities. Such retaliation behaviors are also regarded as attack behaviors (Zhang and Laroche, 2020; Kucuk, 2021). Mostly, they engage in sharing negative emotions on different platforms (Krishnamurthy and Kucuk, 2009; Kucuk, 2018b,^2021^; Zhang et al., 2020). As negative words of mouth are considered harmful to companies, they are aimed at sharing negative information about companies and contain contents of complain, revenge, and disintegration (Istanbulluoglu et al., 2017). Several research studies consider consumer emotions as a major driver for negative words of mouth (Zarantonello et al., 2016, 2018; Hegner et al., 2017; Keiningham et al., 2018; Curina et al., 2020). Sharing negative words of mouth is also considered as a consumer voice against brands to communicate their negative experiences, attitudes, and emotions including brand hate. A recent research study on Indian consumers also found negative words of mouth as a strong means to express their voice as a consequence of brand hate (Sharma et al., 2021). Moreover, the research study also found that negative consumer emotions based on perceptions of unfairness and injustice led to consumer retaliation (Grégoire and Fisher, 2008; Zhang et al., 2020). A recent research study that used the SOR model has confirmed the impact of brand dissatisfaction and brand hate on brand retaliation in United States users of online food platforms (Jabeen et al., 2022). The findings of our studies also imply that consumer dissatisfaction and consumer hate will ignite consumer response in terms of retaliatory behaviors (Noor et al., 2021). Curina et al. (2020) found that brand hate was positively related to NWOM and complaint behavior in a service context. Another study from telecommunication context also found positive impact of brand hate on consumer negative word of mouth (Kurtoğlu et al., 2021).

Research studies also suggest that inward negative emotions result in outward negative emotions, which further ignite consumers to engage in revengeful behaviors (Kurtoğlu et al., 2021). Kurtoğlu et al. (2021) found that consumer regret as an inward emotion was positively related to brand hate, an outward emotion that is further related to negative emotions and a revengeful behavior. This study also adds to evidence from the previous literature and consensus of scholars that consumers’ inward and outward emotions (consumer dissatisfaction and brand hate in this study) are a major source of consumer retaliation in the technology sector of developing countries. Hypotheses 7 and 8 were related to relationship between consumer retaliation and consumer well-being and subjective well-being. The results supported the hypothesis that consumer revenge was negatively related to consumer well-being. This finding implies the consumer response in relation to their external stimuli and internal negative emotions to take deliberate action against a brand to cause damage (Grégoire and Fisher, 2008). In the literature, Grégoire and Fisher (2008) suggested that when consumers have an adverse experience of a brand and develop negative emotions such as brand hate, they engage in certain coping strategies to deal with the hate. The coping strategies are based on whether hate is active or passive.

When a stimulus is strong, it may result in active brand hate, which will further rise the consumer intention to retaliate in the form of taking revenge (Bayarassou et al., 2020). This is also true when people feel a certain level of mismatch between what they expect and what they receive. The mismatch of values and self-concept also stimulates brand hate, which results in negative words of mouth and brand retaliation (Fahmi and Zaki, 2018). A recent study considered that retaliatory behaviors such as consumer complaints attributed to a particular company or a brand reflect decreased consumer well-being related to that particular brand or company (Sirgy, 2020). This is because consumer well-being represents societal well-being and better quality of life of consumers in relation to specific brands. When brands fail to provide stated benefits and fulfill expectations, consumers develop negative emotions, which threaten consumer well-being ultimately (Sirgy, 2020).

## Conclusion

In conclusion, managers of technology brands can enhance consumer well-being by fulfilling consumers’ expectations and symbolic and functional aspects of their brands, and responding to consumer complaints promptly and favorably. Asian consumers seldom share their complaints on stores or brands; rather, they prefer to share a negative experience with peers and friends. This negative word of mouth will damage more than any benefits complaint redressal may offer. Therefore, marketers need to be greatly responsive to consumer complaints to increase consumer well-being.

### Implications

This study also used the SOR model to explain its research framework. This model pertains to cognitive psychology and provides a greater basis for understanding consumer behavior. The basic tenet of this theory states that consumer behavior is the function of some external stimulus and internal processing (Mehrabian and Russell, 1974; Jacoby, 2002). The influence of an external stimulus combined with internal and psychological elements impacts the behavior. This model has been popularly used in environmental psychology, in which certain aspects of environment function as a stimulus (S) to have an influence over a person’s internal feelings (O) and eventually result in a behavior (R) (Eroglu et al., 2001). The stimulus in the SOR model refers to factors in the environment surrounding consumers and arousing consumer emotions (Jacoby, 2002).

In addition to theoretical implications, this study also offers a certain practical implication for producers and marketers of technology brands. First, they need to understand the dynamics of the self-concept of young consumers by understanding the functional and symbolic expectations of brands’ characteristics. Second, they need to redesign and remarket their products to create a fit between what consumers expect and receive to develop better levels of congruence. These efforts will mitigate the sources of band hate and brand dissatisfaction. Advertisers of technology brands should also include clues related to propagating desired values that increase the self-concept of the youth by engaging celebrities if possible. This inculcation of values will also shape newer dimensions of self-concept that can also strengthen their self-congruity, especially symbolic congruity, which will spur positive emotions.

Moreover, product designers need to focus more on functional aspects of technology brands so as to satisfy consumers, as consumer satisfaction also has an impact on consumer well-being. Corporate managers must also realize that the right brand for the right consumer is of paramount importance, particularly in technology-related brands, as young consumers of technology brands are not only strongly aware, but they are also confident and independent decision-makers. Marketers who emphasize on consumer well-being (quality of life marketers) focus on creating brand communities to facilitate self-concept and enhance consumer well-being (Grzeskowiak and Sirgy, 2008). Marketers of technology brands also need to focus on tech brand communities by engaging young consumers and making them ambassadors of the brands. Brands may sponsor major youth-related activities in universities and colleges by holding engaging activities, sponsoring their sports and academic events, and sharing some souvenirs and gifts ascribed with logos and messages of brands to create a connection between them and consumers.

### Limitations and future recommendations

This study has also certain limitations that should be kept in mind before drawing conclusions from this study. The first limitation is regarding the type of sample of this study. This study collected data from young users of technology products, and 72% of the participants fell in the age group of 21–30 years. The Pakistani population is comprised of 68% youths (Bakari and Hunjra, 2018); however, scholars may be interested to know whether any different combination of age groups may have impacted the results or not. Therefore, future researchers may collect data from technology product users of Pakistan having fair representation from various age groups. Alternatively, the results may vary if data are collected from different cultures. This study used functional and symbolic incongruities as antecedents to brand hate and brand dissatisfaction. As this study collected data from technology product users, using TAM models as antecedents to brand hate and dissatisfaction may be another line of inquiry for future researchers (Islam et al., 2020; Garg and Pandey, 2021). This study collected data using self-report questionnaires and had a cross-sectional design. However, efforts were been made to inform the participants of the confidentiality and voluntariness of the data following (Podsakoff et al., 2003 and despite the recently acknowledged practical relevance of cross-sectional design (Spector, 2019). This study fairly assumes that consumer well-being may vary with the passage of time; therefore, longitudinal designs may offer some interesting and maybe curvilinear relationships among self-incongruity, negative brand emotions, anti-branding behaviors, and consumer well-being.

## Data availability statement

The raw data supporting the conclusions of this article will be made available by the authors, without undue reservation.

## Ethics statement

Ethical review and approval was not required for the study on human participants in accordance with the local legislation and institutional requirements. The patients/participants provided their written informed consent to participate in this study.

## Author contributions

All authors listed have made a substantial, direct, and intellectual contribution to the work, and approved it for publication.
